# Locking the GFP Fluorophore to Enhance Its Emission Intensity

**DOI:** 10.3390/molecules28010234

**Published:** 2022-12-27

**Authors:** Joana R. M. Ferreira, Cátia I. C. Esteves, Maria Manuel B. Marques, Samuel Guieu

**Affiliations:** 1 LAQV-REQUIMTE, Department of Chemistry, University of Aveiro, Campus de Santiago, 3010-193 Aveiro, Portugal; 2 LAQV-REQUIMTE, Department of Chemistry, School of Science and Technology, New University of Lisbon, 2829-516 Caparica, Portugal; 3 CICECO-Aveiro Institute of Materials, Department of Chemistry, University of Aveiro, Campus de Santiago, 3010-193 Aveiro, Portugal

**Keywords:** fluorescence, green fluorescent protein, Z/E isomerization, aggregation-induced emission enhancement, difluoroborate

## Abstract

The Green Fluorescent Protein (GFP) and its analogues have been widely used as fluorescent biomarkers in cell biology. Yet, the chromophore responsible for the fluorescence of the GFP is not emissive when isolated in solution, outside the protein environment. The most accepted explanation is that the quenching of the fluorescence results from the rotation of the aryl–alkene bond and from the Z/E isomerization. Over the years, many efforts have been performed to block these torsional rotations, mimicking the environment inside the protein β-barrel, to restore the emission intensity. Molecule rigidification through chemical modifications or complexation, or through crystallization, is one of the strategies used. This review presents an overview of the strategies developed to achieve highly emissive GFP chromophore by hindering the torsional rotations.

## 1. Introduction

Luminescence is described as the spontaneous emission of light from an electronic excited state to the ground state [1]. During the luminescence phenomenon, there are a series of transitions that are described in the Jablonski diagram [2]. In short, after energy absorption, the fluorophore goes from the ground state to an excited state, and when the molecule returns to the ground state, it can emit a photon in the form of fluorescence. The presence of electron-donating and electron-withdrawing groups around a conjugated core can promote a shift in the absorption and emission spectra. When a dye has a highly conjugated π system with a planar structure, the molecules tend to aggregate and establish π–π stacking interactions, which will quench their emission in a phenomenon called aggregation-caused quenching (ACQ) [3]. In contrast, some dyes that are not emissive in solution, or poorly emissive, can become highly emissive when in the solid state, due to the restriction of intramolecular rotations and torsional vibrations [4], in a phenomenon known as aggregation-induced emission (AIE) or aggregation-induced emission enhancement (AIEE) [3].

**Figure 1:**
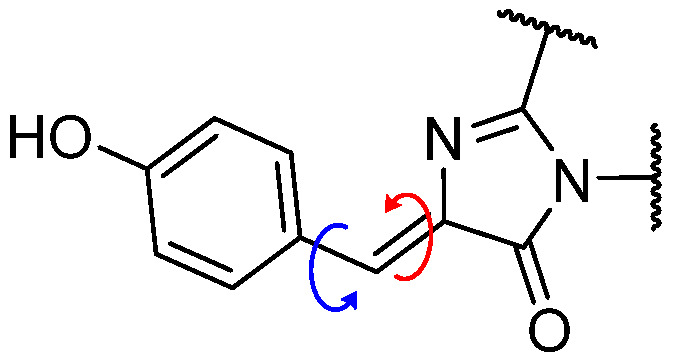
*p*-hydroxybenzylidene-imidazolidinone chromophore responsible for the emission of the GFP.

The Green Fluorescent Protein (GFP) is a light-producing protein from the jellyfish Aequora Victoria [5] with a bright green, fluorescent emission. In 2008, the Nobel Prize in chemistry was attributed to Osamu Shimomura, Martin Chalfie and Roger Tsien for the work developed on the discovery and isolation of the GFP [6]. One advantage is that the GFP can easily be linked to a protein, via genetic modification of an organism. This is particularly useful to monitor the level of expression of a protein of interest, or its localization, via fluorescence measurements. The chromophore responsible for its luminescence is *p*-hydroxybenzylidene-imidazolidinone (*p*-HBDI) (Figure 1) and is formed inside the β-barrel structure during the protein folding, due to the chemical transformation of serine, tyrosine and glycine residues from where the chromophore is formed [7]. However, besides being responsible for GFP emission, the *p*-HBDI by itself is not emissive when isolated in solution. The loss of fluorescence emission of this chromophore is usually attributed to non-radiative decay due to the internal rotations of the molecule, such as the rotation of the aryl–alkene bond (Figure 1, blue arrow) caused by heat [8]. The photoisomerization of the benzylidene double bond (Figure 1, red arrow) can also be responsible for the emission quenching, changing the fluorophore from an emissive conformation (Z isomer) to a non-emissive conformation (E isomer) [9].

The localization of the chromophore inside the β-barrel of the protein blocks its internal rotations and the Z/E isomerization of the carbon–carbon double bond by a hydrogen-bond network [10]. This blocking suppresses the non-radiative decay and promotes the radiative pathway, enhancing the fluorescence emission. Some strategies have been employed to enhance the emission intensity of the GFP chromophore (GFPc), involving different processes and methods, which include crystallization [11], encapsulation in a macrocycle [12], complexation with metals [13] or with boron [9], or chemical modifications [10]. Additionally, modifications of the structure of GFPc have also been performed in an attempt to tune its emission color. 

**Scheme 1:**
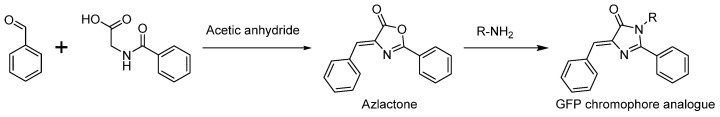
Erlenmeyer–Plöchl azlactone synthesis of a GFPc analogue.

Concerning the synthesis of GFPc analogues, two main strategies have been extensively used: the Erlenmeyer azlactone synthesis and a Knoevenagel condensation. Erlenmeyer–Plöchl azlactone synthesis consists in the condensation of aromatic aldehydes with hippuric acid in acetic anhydride to produce an azlactone [14], then a subsequent reaction with a primary amine leads to the GFPc analogue (Scheme 1) [15].

**Scheme 2:**
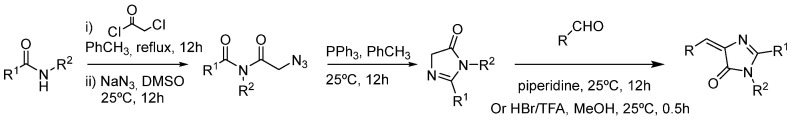
Synthesis of GFPc analogues via Knoevenagel condensation.

Concerning the Knoevenagel condensation applied to the synthesis of GFPc analogues, it involves the formation of the imidazolidine core, followed by a Knoevenagel condensation (Scheme 2) [9].

This review provides an overview of the GFP-inspired fluorophores reported in the past few years, focusing on the different strategies implemented to enhance their emission intensity: crystallization or aggregation, interaction with macromolecules, complexation with boron or metallic cations, chemical modifications, all of them aiming at restricting the torsional vibrations and locking the alkene bond isomerization.

## 2. Fluorescence Enhancement without Structural Modifications

### 2.1. Crystallization and Aggregation-Induced Emission Enhancement (AIEE)

The internal rotations of a chromophore can be blocked when it is in the solid state, as a crystal or an amorphous aggregate, which can lead to the enhancement or induction of the emission, in chromophores that are poorly- or non-fluorescent in solution.

**Figure 2:**
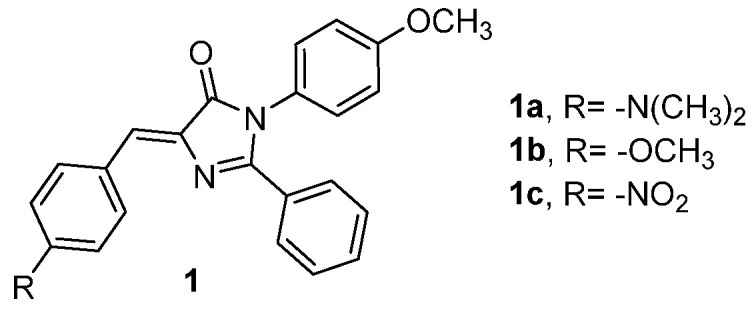
GFPc analogues with multiple phenyl substituents [15].

A family of GFPc analogues has been synthesized using Erlenmeyer azlactone synthesis, followed by a reaction of the obtained oxazolones with *p*-anisidine to give origin to the benzamides ring opening with a final cyclization (chromophores **1a-c**, Figure 2) [15]. The strategy is based on the introduction of rotational aromatic groups around the imidazolidinone core to increase the AIEE phenomena. In solution, the synthesized chromophores present weak emission, due to the relaxation through the Z/E isomerization and torsional vibration of the aromatic rings. This isomerization could be blocked in the aggregate or crystalline state, from where the emission was increased, as expected, and only the presence of the Z isomer was confirmed through the crystal structures [15].

**Figure 3:**
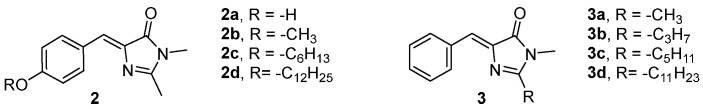
GFPc analogues presenting AIEE properties [10,11].

The crystal packing of a chromophore is considered an important factor to achieve an enhanced emission. The length of the chain attached to the phenolic oxygen influences the crystal packing and the emission properties of the four GFPc derivatives **2** (Figure 3) [11]. Crystals of chromophores **2b**, **2c** and **2d** present AIEE properties. The emission of these chromophores increases along with the alkyl chain length, due to the reduction in the interaction strength between the molecules. The chromophore **2a** did not show fluorescence emission even in the solid state, probably due to the intermolecular hydrogen bonds between the hydroxyl and carbonyl groups [11]. 

The presence of an alkyl chain on the position 2 of the imidazolinone ring (chromophores **3a-d**, Figure 3), demonstrates an effect similar to that described above. Regardless of the length of the alkyl chain, all derivatives are weakly emissive when in solution, but their emission intensity increases when they are in the solid state [10]. Additionally, the solid-state quantum yields increase with the extension of the alkyl chain. This phenomenon could be explained by the changes in the intermolecular arrangements and interactions, which results in weakened intermolecular π–π interactions between the benzylidene–imidazolinone moieties [10].

**Figure 4:**
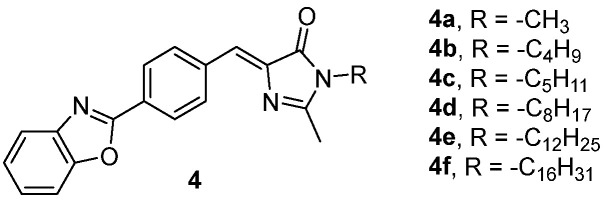
GFPc analogues presenting AIEE properties, combined with ACQ for some of them [16,17].

A family of GFPc containing a 2-phenylbenzoxazole group, and different alkyl chains on the nitrogen in position 3 of the imidazole ring (chromophores **4a-f**, Figure 4) were described as weakly fluorescent in solution, with quantum yields around 0.02, with the exception of **4e** and **4f** which exhibit quantum yields of 0.20 and 0.24, respectively [16,17]. The quantum yield of **4a** does not increase in the crystalline state, while all the other show an AIEE effect with quantum yield of 0.16–0.26 for **4b-d** in the solid state. Derivatives **4e-f** demonstrate a bright fluorescence when adsorbed on a solid support, such as nylon or paper, with a red-shifted wavelength relatively to the DMSO solution, mainly due to the stiffening of the molecules by the adsorption on the solid material [17].

The different behaviors can be rationalized considering the crystal packing of the dyes. The crystal structure of **4a** reveals that it arranges into head-to-tail dimeric structures, with a strong overlap of the benzoxazole of one molecule with the imidazole ring of the other molecule, probably quenching the emission. This overlap is much weaker for the remaining derivatives, preventing the quenching and allowing the increase in their fluorescence through the AIEE effect [16,17]. Additionally, the long alkyl chains, in the case of **4e-f,** seem to increase the fluorescence intensity by separating the molecules [17], which is in agreement with the other results [10]. 

In another article, the polymorphism of **4a** was studied, and the different polymorphs demonstrated to have different photophysical properties [18]. Some polymorphs are non-emissive, but two of them, one containing the pure dye and one solvate, exhibit a strong emission at 502 nm (φ = 0.22) and 582 nm (φ = 0.11), respectively. Molecular motions are restricted in all polymorphs; therefore, all should present AIEE properties, but in some of them, the strong overlap of aromatic rings probably counteracts the AIEE effect with an ACQ effect, quenching the emission [18].

**Figure 5:**
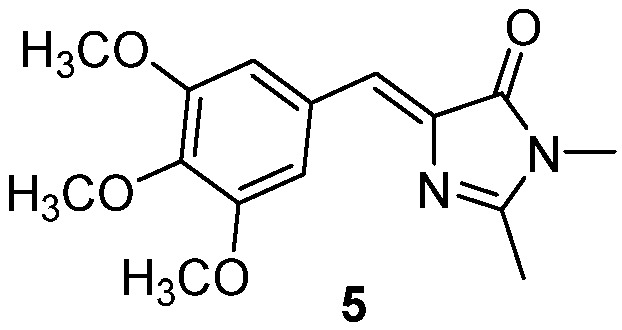
GFPc analogue emitting in the solid state, at different wavelengths depending on the polymorph [19].

The emission of the GFPc **5** (Figure 5) was not detected in solution. In the crystalline state, **5** can organize in five different polymorphs, all of them presenting AIEE properties with quantum yields of 0.02-0.05, due to the restriction of molecular motions. The emission wavelength is different between the polymorphs, varying from a blue emission around 450 nm to a yellow emission around 550nm, as a result of the strength of the π–π interactions between the donor and the acceptor units in the polymorphs [19].

**Figure 6:**
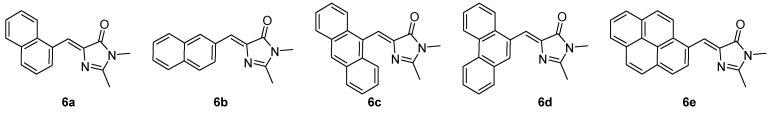
GFPc analogues with large aromatic substituents [20].

In an attempt to rigidify the backbone of the dyes, large aromatic substituents were introduced in replacement of the *p*-hydroxyphenyl ring on the GFPc **6a-e** (Figure 6) [20]. Unfortunately, this did not restrict the intramolecular motions in solution and, consequently, did not increase the emission intensity. However, these modifications induced a red shift of the emission wavelength due to extra π conjugation, and solvent polarity dependence. This shift was also observed in the solid state, but GFPc **6a**, **6c** and **6d** do not present AIEE properties, being only faintly emissive. On the contrary, **6b** crystallized in two polymorphic forms, one of them strongly emissive [20].

**Figure 7:**
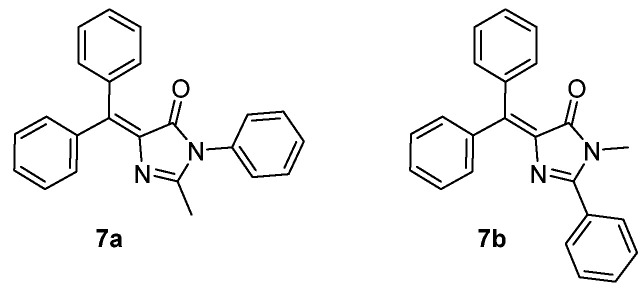
GFPc analogues without Z /E isomerization [21].

The introduction of a diphenylmethylene group in GFPc **7a,b** (Figure 7) was expected to enhance their emission intensity in solution, because it would suppress the possibility of Z/E isomerization (the virtual Z and E isomers are the same molecule). However, they are only faintly emissive in solution. Nevertheless, GFPc **7** presents AIEE properties in the solid state and in frozen solution, due to the restrictions of molecular motions. The crystal packing of GFPc **7a** revealed intermolecular π–π interactions, responsible for the quenched fluorescence [21].

Looking at these examples, we can conclude that many GFPc analogues present AIEE properties and are brightly emissive in the solid state. However, many others are not, and it is difficult to predict the photophysical properties of a GFPc analogue in the solid state. Nevertheless, this approach is limited to the use of the luminescent dyes in the solid state, and many applications require the dyes to be luminescent in solution. 

### 2.2. Supramolecular Hosts

In the GFP, the GFPc is surrounded by the β-barrel, which restricts its torsional vibrations and hinders the isomerization of the phenylene double bond, therefore producing the same effect as aggregation, but preventing ACQ as the dye is isolated. Following a biomimetic approach, supramolecular hosts have been used to encapsulate some GFPc analogues, in an attempt to enhance their emission intensity. 

**Figure 8:**
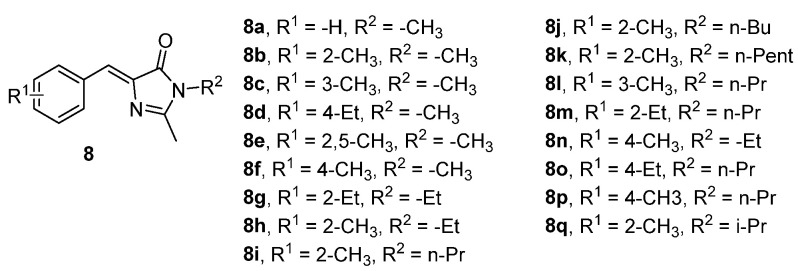
GFPc analogues demonstrating an increased emission intensity in the presence of a deep cavity cavitand [22].

It has been demonstrated that some GFPc analogues (chromophores **8**, Figure 8) could serve as guests inside a deep cavity cavitand (called “octaacid”). These dyes are poorly emissive in solution, but their emission intensity increased dramatically when they enter the cavity of “octaacid”. The GFPc **8k**, bearing the longest alkyl substituent, was the one that demonstrated the greater increase in emission quantum yield, from 1.47% in benzene to 10% in the presence of “octaacid” [22].

**Figure 9:**
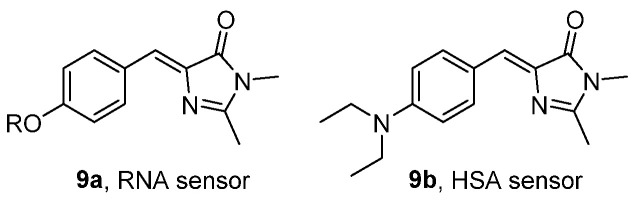
GFPc analogues used as selective fluorescence turn-on molecular probes for RNA and HSA [23].

The fluorescence of a series of GFPc derivatives have been studied in the presence of different biomacromolecules, and these dyes demonstrated that these could be used as small molecular probes (chromophores **9a,b**, Figure 9). The presence of an alkyloxy group in the *para* position of the phenyl ring induced a turn-on fluorescence when in the presence of ribonucleic acid (RNA), while the diethylamino group in the same position induced a selective fluorescence increase in the presence of human serum albumin (HSA). It is likely that the interaction of the dyes with the biomacromolecules stiffen their backbone, thus restricting molecular motions and isomerization [23].

**Figure 10:**
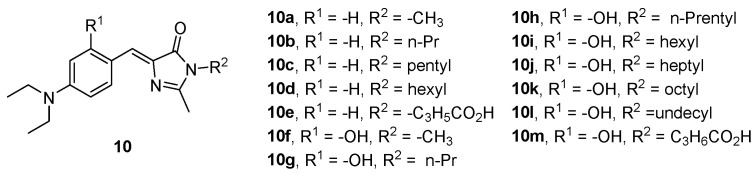
GFPc analogues with enhanced emission intensity in the presence of HSA [24].

Moreover, all GFPc **10** (Figure 10) demonstrated that their fluorescence intensity increases when bonded to HAS; the longer the alkyl chain of R^2^, the more dramatic the enhancement, with chromophore **10k** being the one that showed the best results [24].

**Figure 11:**
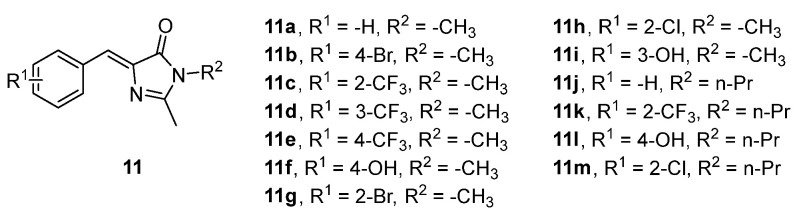
GFPc analogue with enhanced emission when encapsulated in NaCh vesicles [25].

Similar analogues of the GFPc (chromophores **11**, Figure 11) also demonstrated an enhanced emission intensity when encapsulated inside amphiphilic sodium cholate (NaCh), most probably because their torsional motions are restricted in the aggregate cavity. The emission intensity further increases alongside the concentration of NaCh. The authors of this study also proposed that the groups *o*-CF_3_ and *n*-Pr promote the optimal packing in the aggregate cavity, and an additional hydrophobicity, respectively, which results in the emission enhancement of the chromophore [25].

**Figure 12:**
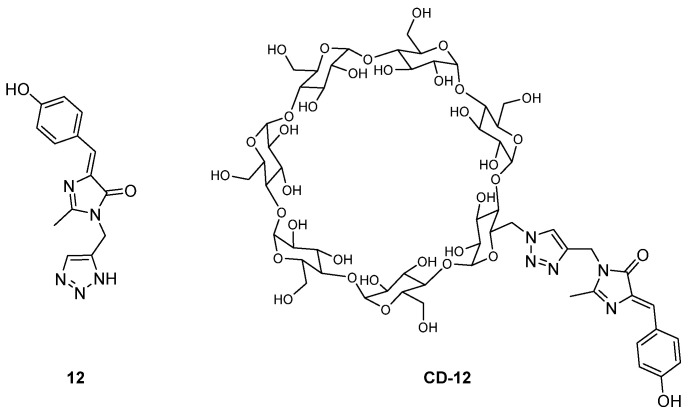
GFPc analogue isolated and attached to a β-cyclodextrin [12].

A β-cyclodextrin could mimic the protein environment, and act as a supramolecular host upon inclusion of a GFPc inside its cavity. The fluorescence intensity of a GFPc analogue (Figure 12) was clearly enhanced when it changed from the isolated chromophore **12** to the chromophore covalently attached to a β-cyclodextrin **CD-12** [12]. Here, the molecular motions of the GFPc, and the double bond isomerization, are blocked by its inclusion inside the cavity of the β-cyclodextrin.

**Figure 13:**
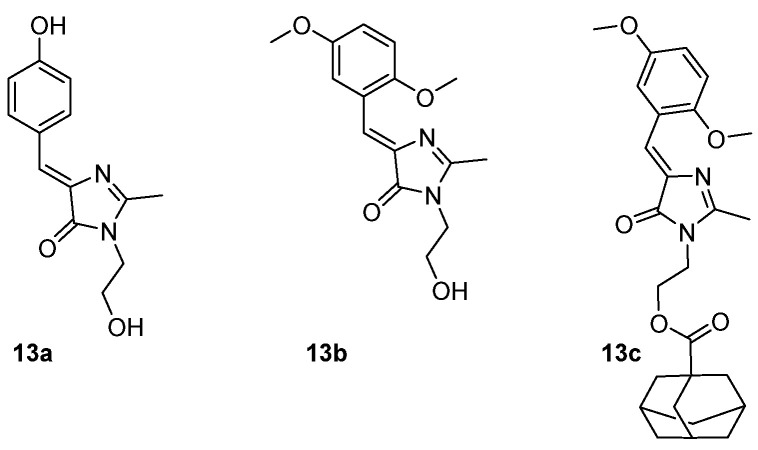
GFPc analogues used to study the supramolecular interaction with cyclodextrin [27].

A family of self-restricted GFPc analogues (chromophores **13a-c**, Figure 13) has been designed to be self-restricting, based on previous report [26], and their emission properties have been studied in solution, in the aggregate state and upon complexation inside a cyclodextrin cavity. In solution, the self-restricted chromophores **13b** and **13c** present higher emission when compared to chromophore **13a**, due to the steric hindrance of the methoxy group, which limits the rotation of the benzylidene ring. GFPc **13c** presents an enhanced emission compared to **13b** probably due to the reduced hydrogen bonding effect [27]. The emission of **13a** and **13b** is quenched in the aggregate state, but **13c** presents an enhanced emission intensity when it aggregates, which can be due to the segregation effect of the adamantyl group. Different β-cyclodextrins derivatives were used to study their effect on the fluorescence of these chromophores. Chromophore **13b** does not seem to interact with the β-cyclodextrins, but GFPc **13c** demonstrated an enhanced emission intensity on the presence of β-cyclodextrins, as the adamantyl group is known to readily enter the cyclodextrin cavity. The complexation with methyl-β-cyclodextrin proved to promote a higher emission [27].

**Figure 14:**
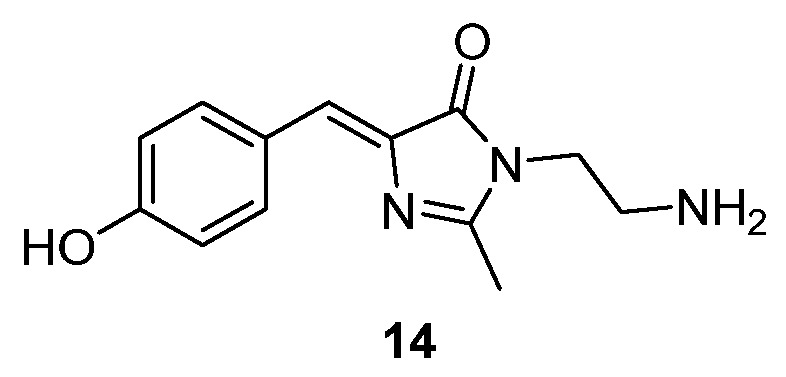
GFPc analogues that can be conjugated with the TMV channel [28].

In a similar approach, the inner surface of the Tobacco Mosaic Virus (TMV) has been used to mimic the GFP β-barrel, as they are quite similar. Chromophore **14** (Figure 14) could be easily conjugated to the glutamate residues of the TMV channel through its amino group. After conjugation, the fluorescence intensity of TMV-**14** exhibited a significant enhancement when compared to the isolated GFPc **14**, with an extra enhancement in the presence of different organic solvents, with DMSO being the one that showed the most dramatic results [28].

**Figure 15:**
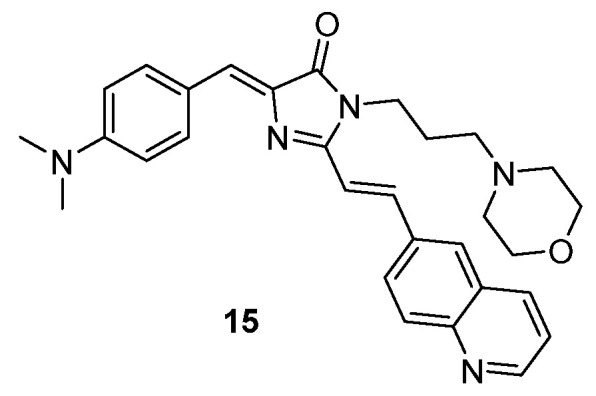
GFPc analogues used as molecular probe for imaging β-amyloids and lysosomes [29].

A GFPc has been modified in order to increase its affinity for β-amyloid fibrils and lysosomes (chromophore **15**, Figure 15). The phenolic hydroxyl group of the GFPc has been replaced by a dimethylamino group, and a quinolone substituent has been introduced, in order to improve the affinity with β-amyloid fibrils. The 3-morpholinopropyl-amino group has been added as a lysosome-targeting group [29]. The GFPc **15** demonstrated to accumulate in the β-amyloids, which mimic the environment of the β-barrel of the GFP, thus turning on the fluorescence. The same effect was observed with lysosomes. Therefore, GFPc **15** allowed the detection of Aβ fibrils and the mapping of viscosity in lysosomes, with fluorescence emissions at 570 nm and 600 nm, respectively [29].

### 2.3. Polymers

**Figure 16:**
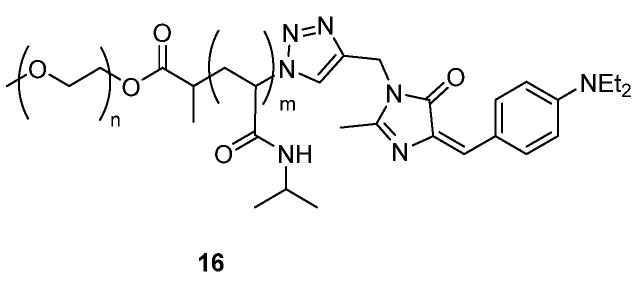
GFPc analogues linked to copolymer [30].

The linking of GFP-inspired chromophores to a polymer chain has been proven effective in terms of enhancing the fluorescence emission. The polymer **16** (Figure 16) was designed by linking a GFPc analogue to the thermo-sensitive copolymer poly(ethylene glycol)-poly(*N*-isopropylacrylamide (PEG-PNIPAM) and exhibited a weak fluorescence at low temperature [30]. Copolymer **16** emits more brightly when above the lower critical solution temperature, which can be explained by the hindrance of the conformational motions of the GFPc due to the self-assembly of the PEG-PNIPAM blocks [30].

**Figure 17:**
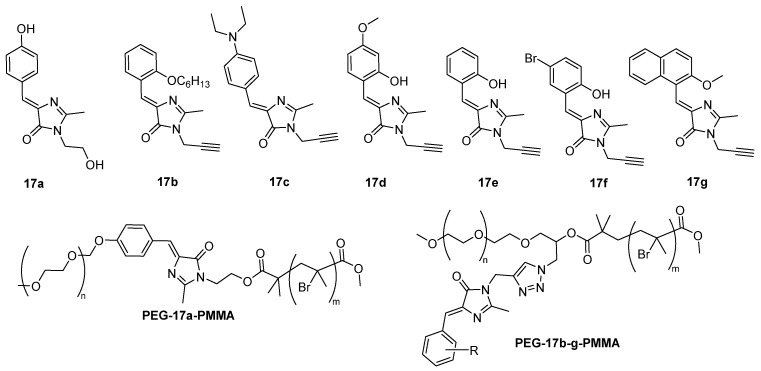
GFPc-copolymer with strong fluorescence after self-assembly into micelles [31,32].

The copolymer can also be built around a single GFPc. For example, the copolymer poly(ethylene glycol)-**17a**-poly(methylmethacrylate) (**PEG-17a-PMMA)** has been grown on GFPc **17a** (Figure 17). Compared to the isolated GFPc **17a**, **PEG-17a-PMMA** demonstrated a red-shifted emission and an increased emission intensity (24 times) after the self-assembly of the GFP-copolymer into micellar aggregates [31]. This can be rationalized by the higher planarity of the chromophore and by the interactions between the chromophore and the copolymer [31]. The GFP-copolymers poly(ethylene glycol)-**17b-g**-poly(methylmethacrylate) (**PEG-17b-g-PMMA)** include one chromophores **17b-g** between the two parts of the copolymer (Figure 17). They also present a brighter fluorescence after assembly into micelles, with different emission wavelengths depending on the GFPc used (**17b-g**), varying between blue, green, yellow and orange [32]. This emission enhancement is related to the length of the hydrophobic chain, and is larger with longer chains, because these chains are able to reduce the intermolecular interactions and inhibit the molecular motions of the chromophore [32].

**Figure 18:**
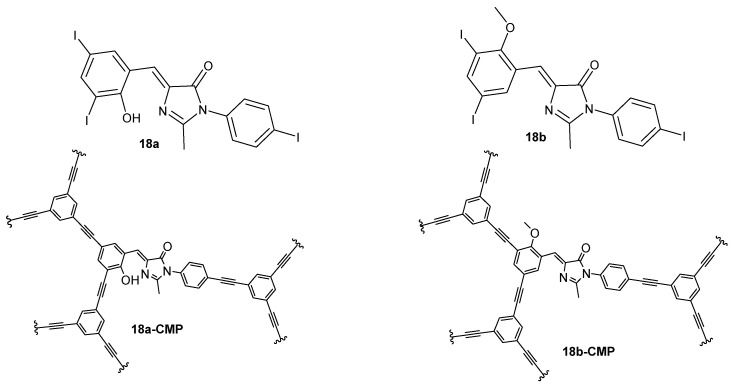
GFPc analogues used in the preparation of porous organic frameworks [33].

The connection of a GFPc with an organic spacer promotes the formation of a three-dimensional porous organic polymer with luminescent properties similar to the GFP. Two conjugated microporous polymers (**18a-CMP** and **18b-CMP**, Figure 18) were prepared from two GFPc analogues (**18a,b**, Figure 18). The crystal structure of **18a** revealed an intramolecular hydrogen bond between the proton of the hydroxyl group and the nitrogen of the imidazole. The quantum yields of the GFPc **18a** and **18b** are very low in methanol, mainly due to presence of the iodine atom which quenches the emission [33]. The molecular motions of the chromophores proved to be restricted in the three-dimensional network, and the emission properties of **18a-CMP** are similar to the GFP [33].

**Figure 19:**
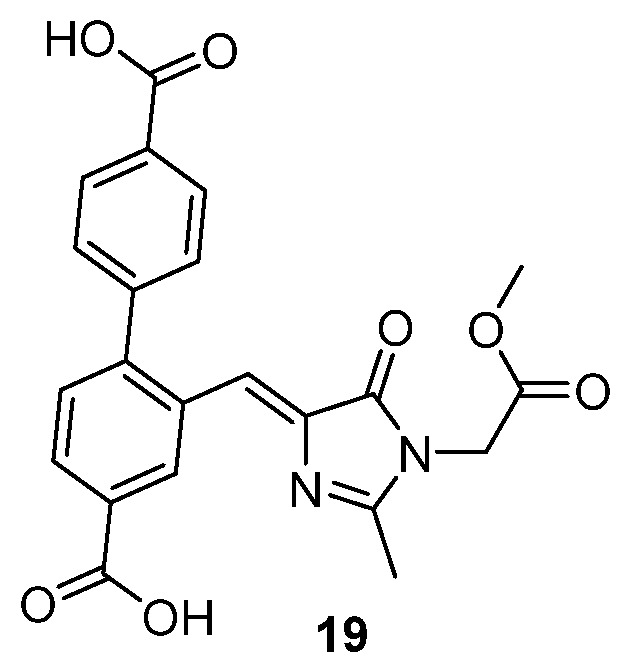
GFPc analogue used as linker in the preparation of a green-fluorescent metal–organic framework [34].

The incorporation of a GFPc into a metal–organic framework was also reported as an alternative to mimic the behavior of the GFPc inside the β-barrel [34]. A metal–organic framework was prepared using GFPc **19** as linker (Figure 19) and zinc (II). It exhibits a green emission similar to the GFP, and the authors demonstrated that the molecular motions of the GFPc are hindered inside the framework [34].

## 3. Fluorescence Enhancement with Structural Modifications

The physical rigidification of the GFPc backbone, through solidification or encapsulation, proved to be a valid strategy to increase its emission intensity. However, another approach is needed if the GFPc is to be used in solution. For this, the skeleton of the dye can be modified to hamper or block the isomerization of the benzylidene double bond.

### 3.1. Intramolecular Hydrogen Bond

The introduction of a strong intramolecular hydrogen bond in GFPc analogues is a simple strategy to hinder the torsional rotations, and the double bond isomerization, responsible for the radiationless deactivation [35]. 

**Figure 20:**
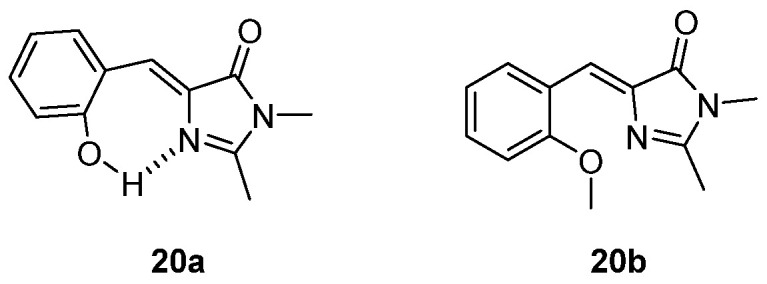
GFPc analogues demonstrating that the intramolecular hydrogen bond increases the emission intensity in solution [35].

In order to demonstrate the validity of this approach, an isomer of the GFPc with the hydroxyl group located on the ortho position of the phenyl ring instead of the para position (chromophore **20a**, Figure 20) has been prepared. A slight enhancement of the quantum yield is observed for GFPc **20a** compared to GFPc **20b**, in which the intramolecular hydrogen bond is absent. The presence of the intramolecular hydrogen bond is supported by the crystal structure of **20a**. These results suggest that this intramolecular hydrogen bond blocks the torsional vibrations and / or the isomerization, thus increasing the emission intensity [35].

**Figure 21:**
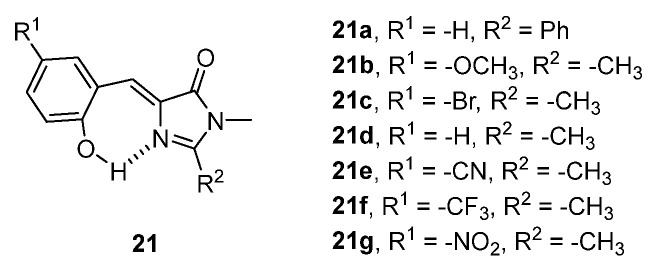
GFPc analogues with enhanced emission intensity due to the intramolecular hydrogen bond [36].

A family of GFPc analogues (chromophores **21a-g**, Figure 21) with a seven-membered ring hydrogen bond, also demonstrated that this intramolecular hydrogen bond is important to hinder the nonradiative deactivation associated with the rotation of the exocyclic double bond [36]. Moreover, these dyes presented an Excited-State Intramolecular Proton Transfer, which shifts the emission towards longer wavelengths.

**Figure 22:**
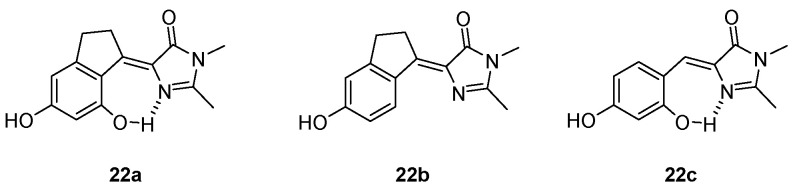
GFPc analogues presenting a hydrogen bond and/or a cyclic substituent [37].

Another example of seven-membered ring hydrogen bonds is presented in the family of chromophores **22a-c** (Figure 22), where it was demonstrated that chromophore **22a** exhibits an enhanced fluorescence (φ=0.18 in toluene) compared to the chromophores **22b** and **22c** [37]. Therefore, it seems that combining an intramolecular hydrogen bond with a cyclic substituent around the double bond is even more efficient to lock the isomerization.

**Figure 23:**
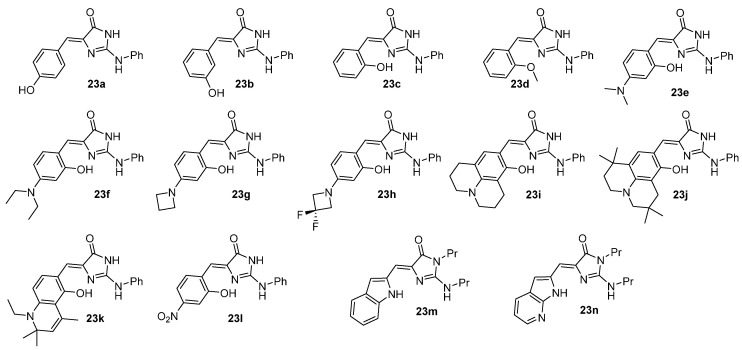
GFPc analogues with a benzimidazole ring substituted with nitrogen, and intramolecular hydrogen bond [38,39].

Changing the substitution of the imidazolone ring with the introduction of heteroatoms is rare but can be promising. For example, the GFPc analogues **23a-n** contain a 2-(phenylamino)imidazolone core (Figure 23). The introduction of an aminophenyl substituent and the repositioning of the hydroxyl group into position 2 of the benzylidene moiety, in GFPc **23c,e-l**, to allow a strong intramolecular hydrogen bonding led to an enhanced emission intensity, an increased Stoke shift (up to 185 nm) and a considerable red shift, when compared with the GFPc **23d** which lacks the hydrogen bond. The addition of a dialkylamino substituent on the benzylidene ring led to a two orders of magnitude enhancement in the quantum yields. Furthermore, an enhanced two-photon cross-section was observed for compounds **23f-k**, which is relevant for certain neurobiological applications [38]. 

Following a similar approach, GFPc **23m,n** include an heteroatom on the imidazolone core, and a strong intramolecular hydrogen bond (Figure 23). These GFPcs demonstrated higher quantum yields than the GFPc lacking the hydrogen bond. Unexpectedly, the GFPc **23n** with an aza-indole substituent shows a much higher emission intensity than the indole analogue **23m** [39]. Further derivatization of these GFPc analogues included hydrophilic groups, so that the dyes can be used for biological imaging.

### 3.2. Boron Complexes

These results demonstrated the intramolecular hydrogen bond can somehow block the isomerization, but that it is not enough to substantially increase the emission intensity. The substitution of the hydrogen bond by a boron complex has been attempted to overcome this limitation.

**Figure 24:**
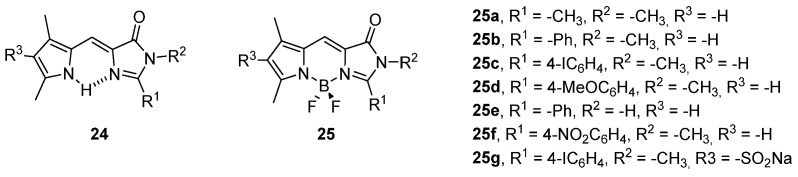
GFPc analogues with a boron complex, demonstrating higher emission intensity than the hydrogen-bonded precursors [9].

The hydrogen-bonded GFPc analogues **24** (Figure 24) emit faintly in solution, with very low quantum yields, demonstrating that the hydrogen bond is not very efficient to block the isomerization of the double bond. They were transformed into a hybrid between a BODIPY and a GFPc by reaction with trifluoroboron etherate, forming the boron complexes **25a-g**. This modification proved to efficiently restrict the rotation and isomerization of the GFPc, and consequently, block the non-radiative decay [40]. Indeed, the GFPc **25a-g** exhibited higher quantum yields, around 0.80-0.89 in methanol, when compared to the precursors **24.** Only GFPc **25f** demonstrated a lower quantum yield (0.0004 in methanol and 0.53 in hexanes), probably due to the presence of the nitroaryl substituent, which may quench the emission [9]. The absorption and the emission wavelengths of all GFPc **25b-g** are quite similar, with exception of derivative **25a**, probably due to the substitution with a methyl group instead of an aryl group [9].

**Figure 25:**
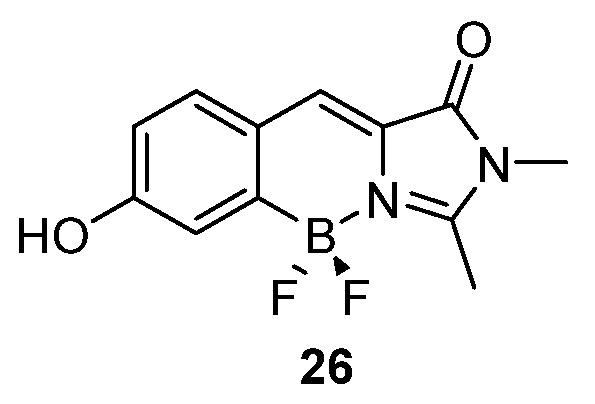
GFPc analogue with the Z/E isomerization blocked by intramolecular interaction [41].

Instead of using a boron complex, the boron can also be covalently linked to the aromatic ring of the benzylidene moieties, forming a donor-acceptor bond with one nitrogen of the imidazole ring (chromophore **26**, Figure 25). This interaction would also lock the isomerization of the benzylidene double bond, resulting in an increase in the emission intensity [41,42,43]. Chromophore **26** presents absorption and emission spectra similar to the GFPc. However, its quantum yield is much higher (φ = 0.73 in acetonitrile), similar to the quantum yield of GFP (φ = 0.60), as a result of the stiffening of the chromophore [41].

**Figure 26:**
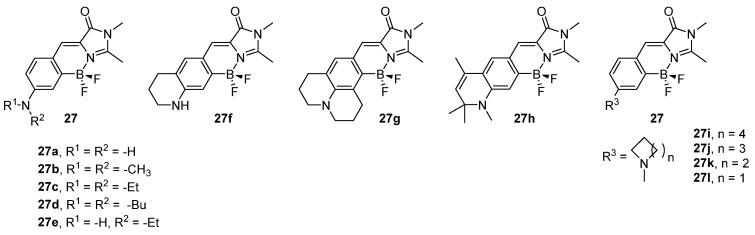
GFPc analogues locked with intramolecular interaction, used to study the effect of the cyclic substituents [40,44].

Based on this result, a family of modified GFPc (chromophores **27**, Figure 26) was designed [40,44]. The modifications performed on these derivatives also promote an increase in their quantum yield, ranging from 0.30 to 0.77 in acetonitrile. These chromophores present a solvent-dependent quantum yield. The introduction of a rigid cycloalkyl substituent on the aromatic amino group of chromophores **27f-h** avoids the twisting of the amino group, promoting an increase in the quantum yield [44]. These modifications of rigidity also induced a red shift in the absorption and emission spectra, compared with the acyclic analogues **27a-d** [44]. 

**Figure 27:**
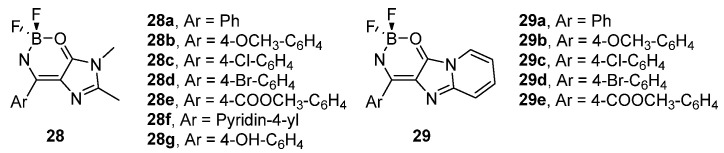
GFPc analogues with a boron complex blocking the Z/E isomerization [45].

This approach to block the isomerization of benzylidene double bond uses a link between the phenyl ring and the core of the imidazolone. Another approach has been attempted, using a boron complex formed between the carbonyl of the imidazolone and an amino group introduced on the double bond of the benzylidene (chromophores **28** and **29**, Figure 27) [45]. The difluoroborate complex efficiently blocks the Z/E isomerization of the double bond and, as expected, the quantum yield increases up to 0.60. These GFPcs also show solvent-dependent quantum yield [37]. Contrary to what has been observed with other GFPc containing a boron complex, the absorption spectra of chromophores **28** and **29** are blue-shifted relatively to the free ligand (without boron), while the emission spectra are red-shifted. The difference in their absorption spectra is due to the nitrogen(3)–boron coordination of the imidazole ring [45].

**Figure 28:**
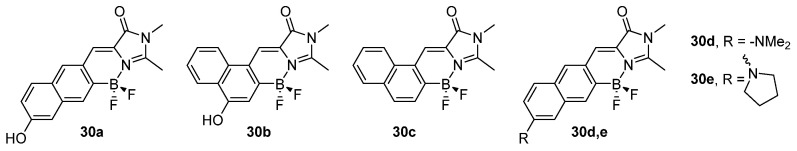
GFPc analogues with a more conjugated backbone [46].

In order to tune the absorption and emission toward longer wavelengths, a family of conformationally locked GFPc containing the naphthalene ring has been prepared (chromophores **30**, Figure 28). This modification leads to an increase in the Stokes shift, up to 100 nm, and to a dramatic bathochromic shift of the absorption and emission maxima when compared to the corresponding GFPc with only one benzene ring (GFPc **26**, Figure 25) [46].

**Figure 29:**
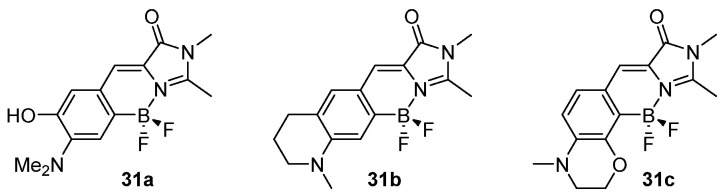
GFPc analogues with solvent-dependent quantum yield (**31a**) or not (**31b,c**) [47].

The peripheral substituents can also have a dramatic effect on the photophysical properties of these dyes. A GFPc analogue locked by a boron complex has been functionalized with a dimethylamino substituent (**31a**, Figure 29). The quantum yield of this dye depends heavily on the solvent polarity, ranging from 0.02 in water to 0.69 in toluene, thus being a potential polarity sensor. On the contrary, when the motions of the amino group are restricted by introduction of a cyclic substituent (**31b** and **31c**), the quantum yield is similar in all solvents (0.60 to 0.95), with a much lower dependence of the polarity [47].

### 3.3. Metal Complexes

**Figure 30:**
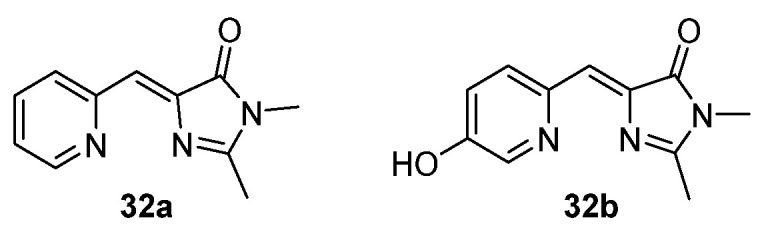
GFPc analogues with an increased emission intensity when complexed with Zn (II) [41,48].

The previous section illustrates the increase in the emission intensity of GFPc when the Z/E isomerization is blocked by a boron complex. Metallic cations can also be chelated by bidentate ligands, and fluorophores have been designed to see their emission modified upon complexation, thus turning them into sensors. This approach has also been developed with GFPc analogues, changing benzyl for 2-pyridyl, forming the bidentate ligand **32a** (Figure 30), with which metallic cations can bind [48]. The chromophore **32a** is weakly fluorescent in solution, and its emission is blue-shifted and quenched when the pH decreases. The fluorescence is also quenched upon addition of metallic cations, with the exception of Zn(II), Cd(II) and Pb(II), which induce an enhancement of the emission intensity 48]. This emission enhancement is rationalized by the locking of the Z/E isomerization upon complexation, with the condition that the metallic cation does not quench the emission.

In another report, the chromophore **32b** (Figure 30) has been demonstrated to complex reversibly with Zn^2+^, producing a bathochromic shift both in the absorption and emission spectra, and its fluorescence also increased when compared to the Zn^2+^ free ligand [41].

**Figure 31:**
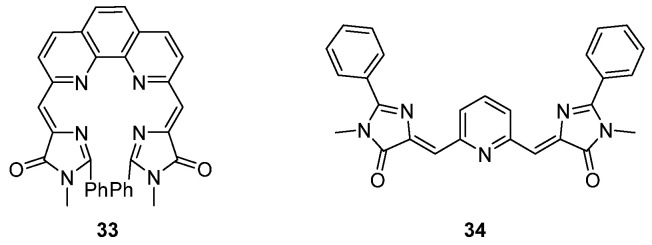
GFPc analogues for the detection of metallic cations [13,49].

Chromophore **33** (Figure 31) was demonstrated to be highly selective to Zn^2+^. The emission spectra of the Zn^2+^ complex is blue-shifted relatively to the free ligand, and the emission intensity is enhanced, but this does not happen with other metal ions [49]. When the pH of the solution decreases, the emission of the complex is quenched. The GFPc **34** (Figure 31) presents similar features, but a different complexing pocket [13]. It also demonstrated an emission enhancement at 474 nm in the presence of Zn^2+^ ions, probably due to the blocking of the aryl–alkene bonds [13]. Additionally, the emission of the complex is also quenched at lower pH, in accordance with previous reports [48,49]. In the presence of Hg^2+^ ions, GFPc **34** changes from its characteristic yellow color (400 nm) to a pink color (550 nm); however, its emission remains undetectable. 

**Figure 32:**
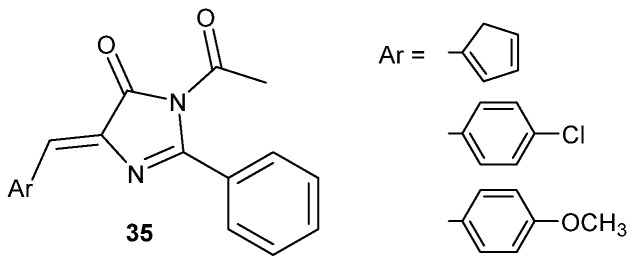
GFPc analogues with a chelating pocket selective for cobalt cations [6].

The GFPc analogues **35 (**Figure 32) presents a different complexing site, introduced by the ring-opening of the azlactone ring with ammonium acetate, followed by a cyclization to obtain the imidazole derivative which was finally reacted with acetic anhydride [6]. It demonstrated good chelation ability toward cobalt, with an enhanced emission intensity upon complexation. 

**Figure 33:**
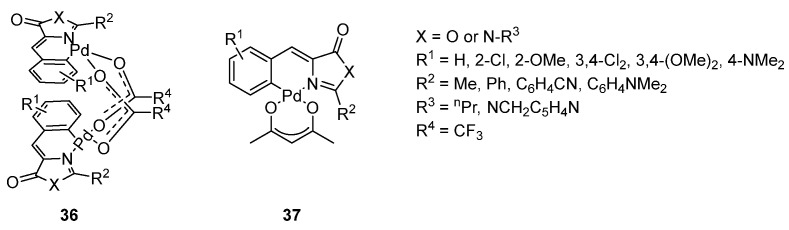
GFPc analogues locked with palladium [50].

Palladium can also be used to block the isomerization of the GFPc, in a manner similar to the boron complexes presented before. For this, the GFPc **36** and **37** have been prepared (Figure 33), starting from azlactones or imidazolones, by orthopalladation. The complexes lead to a rigidification of the GFPc analogues and promoted significant modifications in the absorption and emission wavelengths relative to the free ligand. However, these complexes did not demonstrate enhanced quantum yields, demonstrating that the restriction of the isomerization is not always a sufficient condition to restore the luminescence of the GFPc [50]. 

### 3.4. Covalent Modifications

The restriction of the Z/E isomerization through covalent bonds that limit the rotational motions has also been studied. Different approaches have been explored, such as introducing a cyclic pattern on the benzylidene moiety, and introducing bulky substituents or particular push–pull combinations on the aromatic ring, that would hamper the isomerization.

**Figure 34:**
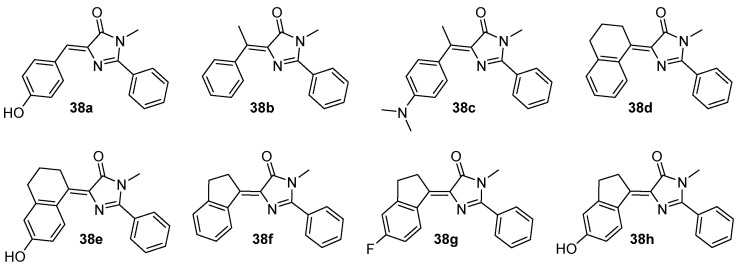
GFPc analogues with isomerization restricted by a cyclic pattern [51].

In the family of GFPc analogues **38a-h** (Figure 34), rotational motions are restricted by the introduction of a cyclic pattern [51]. The locked chromophores **38d-h** demonstrated higher emission intensity than the unlocked **38a-c**, in both solution and solid state. However, even in solution the locked chromophores **38d-h** exhibited a low fluorescence intensity, which indicates that the rotational motion is not the only non-radiative pathway responsible for the loss of fluorescence [44]. This observation had been supported by the study of the crystal structure of chromophores **38** [18].

**Figure 35:**
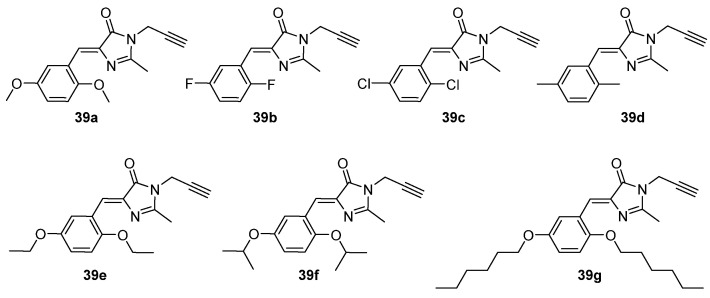
GFPc analogues with their isomerization hampered by benzylidene substituents [26].

The effect of the substituents of the benzylidene ring on the emission of the GFPc has been studied, as they are expected to hamper the isomerization of the double bond. The GFPc **39a** (Figure 35) bear two methoxy groups on the benzylidene ring and exhibits a bright emission in solution. However, if the positions of these methoxy groups are different, the emission is much lower [26]. Additionally, when the substituents are changed to electron-withdrawing or less bulky groups (**39b-d**), the emission is also quenched. The methoxy groups can nevertheless be changed to other ether groups, and GFPc **39a,e-g** demonstrate emission quantum yields of 10.1%, 11.1%, 11.4% and 12.7%, respectively. From these results, the authors concluded that the emission quantum yield increases with the increase in the steric hindrance of the substituents, when they are electron-donating and on the positions 2 and 5 of the aromatic ring. 

**Figure 36:**
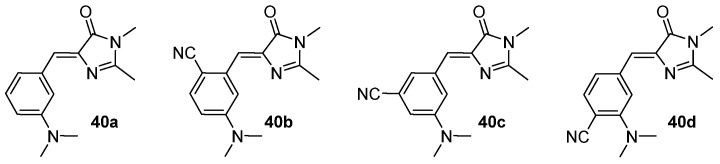
GFPc analogues structurally unconstrained presenting high quantum yields in solution [52].

The effect of the electron-donating or withdrawing character of the substituents has also been studied. For this, a GFPC analogue has been prepared with a dimethylamino group in the meta position of the benzylidene ring, and the position of a cyano group has been varied (chromophores **40a-d**, Figure 36). All of them emit brightly in solution, especially **40b** with a quantum yield of 0.60 in hexane [52]. These GFPcs also demonstrated a dramatic solvatochromism, both for the absorption and emission, with their emission shifting from ca. 480 nm in hexane to ca. 600 nm in acetonitrile. 

## 4. Conclusions

This review summarizes different approaches used to restore the emission intensity of the GFPc outside the protein. The main strategy is to block the torsional rotations and the isomerization of the benzylidene double bond, responsible for the non-radiative decay in solution. Physical methods, such as crystallization or encapsulation, are effective and allow the development of light-up sensors. Chemical methods rely on the modification of the structure of the GFPc, by introducing substituents that restrict or lock the isomerization, thus restoring the emission intensity in solution. These modifications can be permanent, or only light up the GFPc in the presence of a metal ion, in this case forming a sensing probe. Nevertheless, locking the benzylidene double bond of the GFPc is not always enough to enhance its emission intensity. Due to the versatility of its synthesis, and the high number of substituents that can be modified, the GFPc is highly tunable, and many analogues can be prepared to tune their physical, chemical and luminescent properties. All the results presented here demonstrate the high potential of the GFPc for applications in biological imaging, sensors and luminescent materials.

## Data Availability

Not applicable.
